# Supplementary material for: Thrombectomy for medium-sized cerebral vessel occlusion: Size does matter
Source: Eur Stroke J. 2025 Sep 7:23969873251376862. Online ahead of print. doi: 10.1177/23969873251376862 (PMC12417459; doi:10.1177/23969873251376862)

**Supplemental files**

**Supplemental methods**

List of CT scanners

Siemens Definition Edge, Siemens Definition Flash, Siemens Definition AS 63, Siemens Definition AD ,GE Revolution EVO, GE Revolution HD, Toshiba Aquilion Prime 80.

List of used thrombectomy devices

Stent retrievers

Embotrap 5x33mm, Embotrap II 5x33mm (Johnson Johnson: recommended minimum vessel diameter 1,5mm). Trevo XP Provue 3x20mm, 4x20mm, 4x30mm, 6x25mm (Stryker: recommended minimum vessel diameter 2,5mm), Solitaire X 4x40mm (Medtronic: recommended minimum vessel diameter 1,5mm), Solitaire platinum 4x20mm, Solitaire X 6x24mm, (Medtronic: recommended minimum vessel diameter 2mm), Solitaire Platinum 6x40mm (Medtronic: recommended minimum vessel diameter 3mm), NeVa 4x30mm (Mermaid Medical: recommended minimum vessel diameter 2mm), Aperio (Acandis: recommended minimum vessel diameter 1,5mm), Aperio 4,5x30mm (Acandis: recommended minimum vessel diameter 2mm).

Aspiration catheters

AXS Catalyst 5 (Stryker: outer diameter 1,76mm), AXS Catalyst 6 (Stryker outer diameter 1,81mm), AXS Catalyst 7 (Stryker: outer diameter 2,08mm), Sofia Plus (Terumo: outer diameter 2,1mm), ACE 68 CAT (Penumbra: outer diameter 2,03mm), Flowgate (Stryker: outer diameter 2,7mm)

**Supplemental table 1. Vessel size according to sex.**

| **Vessel metrics** | **Female (n=69)** | **Male (n=77)** | **p-value** |
| --- | --- | --- | --- |
| DSA_occluded M2, mm | 1.7 (1.5-1.8) | 1.7 (1.6-1.9) | 0.050 |
| DSA_ipsilateral M1, mm | 2.3 (2.1-2.5) | 2.4 (2.2-2.5) | **0.041** |
| CTA_occluded M2, mm | 1.6 (1.5-1.8) | 1.8 (1.6-1.9) | **0.034** |
| CTA_ipsilateral M1, mm | 2.3 (2.1-2.5) | 2.4 (2.2-2.5) | 0.150 |
| CTA_contralateral M1, mm | 2.3 (2.1-2.5) | 2.4 (2.2-2.6) | 0.053 |

Data are presented as median and IQR. Significant p-values are bolded. DSA, digital subtraction angiography; CTA, computed tomography angiography.

**Supplemental table 2.** Baseline characteristics of M2-occlusion patients not treated with EVT.

| **Variable** | **n=32 missing for 2** |
| --- | --- |
| Age, years | 72 (66-80) |
| Female sex, n (%) | 14 (43.8) |
| pre-stroke mRS, points | 0 (0-1) |
| LSW to Arrival, min | 139 (72-500) |
| Arrival to puncture, min | 67 (57-91) |
| NIHSS prior to EVT, points | 12 (7-15) |
| ASPECTS, points | 9 (8-10) |
| IVT, n (%) | 17 (53.1) |
| **History of** |  |
| Ischaemic stroke, n (%) | 7 (21.9) |
| TIA, n (%) | 3 (9.4) |
| CAD, n (%) | 7 (21.9) |
| Atrial fibrillation ~~fib~~, n (%) | 8 (25.0) |
| Hypertension, n (%) | 20 (62.5) |
| Diabetes, n (%) | 7 (21.9) |
| **TOAST** |  |
| LAA, n (%) | 10 (31.3) |
| CE, n (%) | 17 (53.1) |
| other, n (%) | 1 (3.1) |
| insufficient/unknown, n (%) | 4 (12.5) |

Unless otherwise stated, data are presented as median and IQR. mRS, modified Rankin Scale; LSW, last seen well; NIHSS, National Institutes of Health Stroke Scale; EVT, endovascular thrombectomy; ASPECTS, Acute Stroke Prognosis Early CT Score; IVT, intravenous thrombolysis; TIA, transient ischaemic attack; CAD, coronary artery disease; LAA, large artery disease; CE, cardioembolism.

**Supplemental table 3.** CTA and DSA characteristics of M2-occlusion patients not treated with endovascular thrombectomy.

| **Variable** | **n=32 missing for 2** |
| --- | --- |
| **CTA** | |
| ipsilateral M1, mm | 2.30 (2.20-2.60) |
| contralateral M1, mm | 2.30 (2.20-2.50) |
| occluded M2, mm | 1.50 (1.30-1.80) |
| **DSA** | |
| MeVo-M-TICI 0-1, n (%) | 25 (80.7) |
| ipsilateral M1, mm | 2.40 (2.15-2.50) |
| occluded M2, mm | 1.50 (1.40-1.70) |
| non-dominant M2, n (%) | 17 (53.1) |

Unless otherwise stated, data are presented as median and IQR. CTA, computed tomography angiography; DSA, digital subtraction angiography.

**Supplemental table 4.** Outcomes of M2-occlusion patients not treated with endovascular thrombectomy~~.~~

| **Variable** | **n=32 missing for 2** |
| --- | --- |
| **24h** | |
| NIHSS, points | 5 (2-11) |
| ASPECTS, points | 7 (7-8) |
| **haemorrhage** | |
| any ICH, n (%) | 7 (21.9) |
| ECASS II, n (%) | 3 (9.4) |
| SITS, n (%) | 1 (3.2) |
| **3 months** | |
| mRS 5-6, n (%) | 7 (21.9) |
| mortality, n (%) | 6 (18.8) |

Unless otherwise stated, data are presented as median and IQR, NIHSS, National Institutes of Health Stroke Scale; ASPECTS, Acute Stroke Prognosis Early CT Score; ICH, intracerebral haemorrhage; ECASS II, European Cooperative Acute Stroke Study II; SITS, safe Implementation of Treatments in Stroke; mRS, modified Rankin Scale

**Supplemental figure 1. Distribution of modified Rankin Scale including patients not treated with endovascular treatment (EVT).**


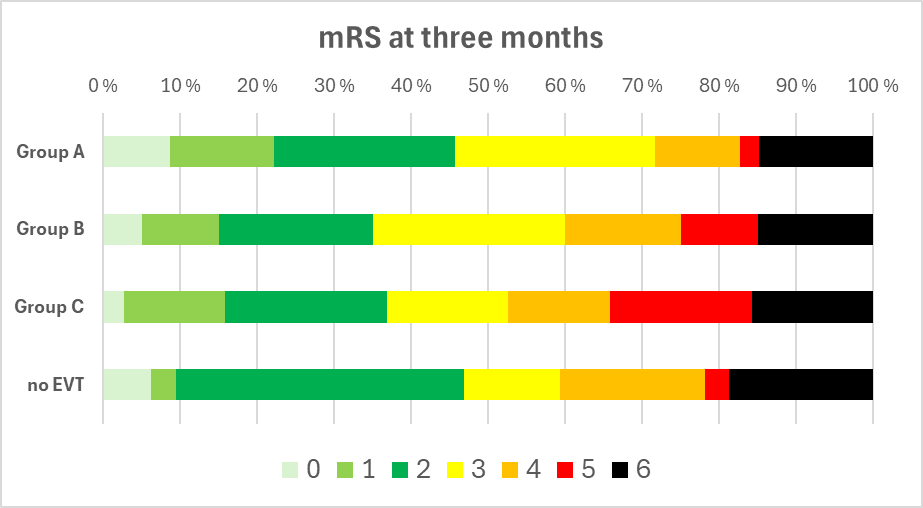

Supplement: sj-docx-1-eso-10.1177_23969873251376862 – Supplemental material for Thrombectomy for medium-sized cerebral vessel occlusion: Size does matter [file sj-docx-1-eso-10.1177_23969873251376862.docx]
