# Supplementary figures and images for: Thrombectomy for medium-sized cerebral vessel occlusion: Size does matter
Source: Eur Stroke J. 2025 Sep 7:23969873251376862. Online ahead of print. doi: 10.1177/23969873251376862 (PMC12417459; doi:10.1177/23969873251376862)

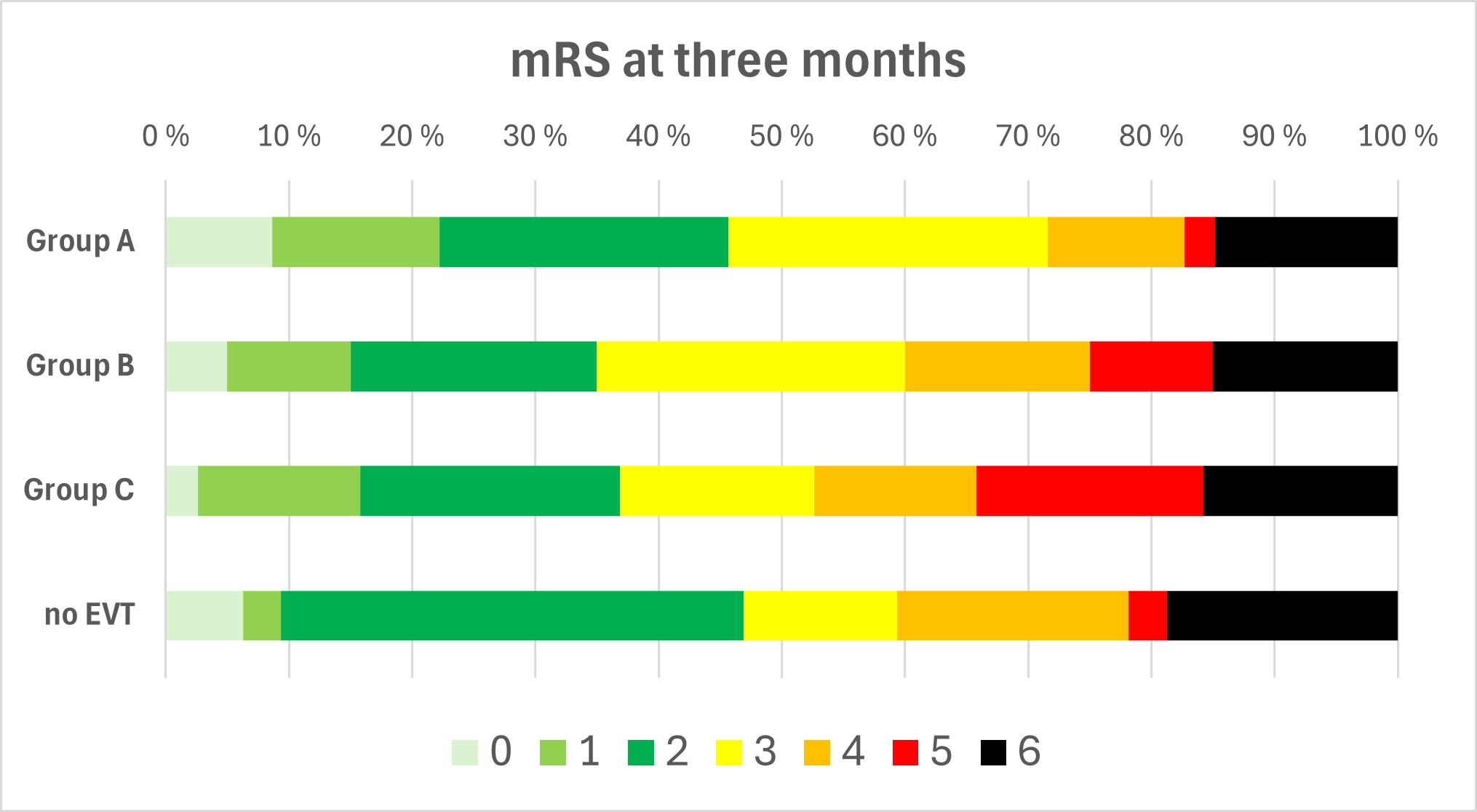

Supplement: sj-jpg-2-eso-10.1177_23969873251376862 – Supplemental material for Thrombectomy for medium-sized cerebral vessel occlusion: Size does matter [file sj-jpg-2-eso-10.1177_23969873251376862.jpg]
